# The behavior of tunisians during the lockdown of COVID-19

**DOI:** 10.1192/j.eurpsy.2021.806

**Published:** 2021-08-13

**Authors:** R. Jomli, A. Sahbani, H. Jemli

**Affiliations:** 1 Faculty Of Medicine Of Tunis, university of tunis elmanar, tunis, Tunisia; 2 Sociology, faculty of human sciences, tunis, Tunisia

**Keywords:** COVID19, Tunisia, behavior, general confinement

## Abstract

**Introduction:**

The general confinement in Tunisia in the covid-19 pandemic is a new event for the Tunisian society with economic, social and psychological repercussions.

**Objectives:**

To evaluate the behavior of Tunisians during the general confinement of 2020.

**Methods:**

descriptive and analytical study through a questionnaire sent online under the model of “google forms”.

**Results:**

Our sample is composed of 500 people, mostly women, with an average age of about 40 years, an average to good economic level, and a secondary and higher education level. In the Tunisian family, the most discussed topic during the lockdown is the covid-19 and its evolution in the world and the country. The most avoided subject is the behavior of neighbors. The Tunisian’s main sources of information on covid-19 are television and social networks. The behavior most adopted to avoid contamination is hand washing. Only 2/3 of the group applied the measures announced by the government. A quarter of our sample spent more than 5 hours in front of the computer screen or smartphone. During confinement the most important behaviors are smoking, eating, doing nothing and playing cards. Only 10% of our sample have plans for next year.

**Conclusions:**

For our sample, covid-19 has greatly transformed the behavior in daily life which has become dominated by anxiety and fear of contamination.

